# Ultrasonically Estimated Bladder and Detrusor Weights in Patients with Post-Void Residual Urine

**DOI:** 10.1590/S1677-5538.IBJU.2025.0126

**Published:** 2025-03-29

**Authors:** Aderivaldo Cabral Dias, Maria Laura Regis Cabral Dias, Guy Grebot

**Affiliations:** 1 Universidade de Brasília Hospital de Base Faculdade de Ciências Médicas Brasília DF Brasil Unidade de Urologia, Hospital de Base. Faculdade de Ciências Médicas, Universidade de Brasília, DF, Brasil; 2 Universidade de Brasília Faculdade de Engenharia Brasília DF Brasil Faculdade de Engenharia, Universidade de Brasília, DF, Brasil; 3 Universidade de Brasília Departamento de Matemática Brasília DF Brasil Departamento de Matemática, Universidade de Brasília, DF, Brasil

To the editor,

The recently published article by Huseynov and associates (1) used both urinary nerve growth factor and ultrasonically measured bladder wall thickness (BWT) to assess the response to urotherapy and antimuscarinic treatment in children with overactive bladder. These authors acknowledge that the main limitation to the use of BWT is its dependence on bladder filling. One solution to overcome this limitation, derived from the observation that BWT would not significantly change after a given intravesical volume (2), is to measure BWT with a quasi-standardized intravesical volume (250 ml, say). This, however, only works in normal weight bladders: with hypertrophy, BWT will continue to change with bladder filling.

A more anatomically and physiologically sound solution is to use ultrasonically estimated bladder weights (UEBW), either by 3D ultrasound imaging, which is costly and not widely available; or by calculating the volume of tissue enclosed by two concentric spheres (a spherical shell), as proposed by Kojima et al (3). Although Kojima experimentally demonstrated that this method strongly correlates with the actual weight of the organ (R = 0.97), it also requires either complete bladder emptying, thus excluding patients with high post-void residuals; or bladder catheterization, which defeats the purpose of UEBW calculation as a non-invasive diagnostic test.

Our solution to this conundrum is to recognize that in an idealized spherical bladder, the voided volume is the difference between pre-void and post-void intravesical volumes, determined by the inner sphere's radius before and after voiding (*r*_pre_*, r*_post_), that is:


(1) $$V_{void}=\frac{4}{3}\pi \left(r_{pre}^{3}-r_{post}^{3}\right)$$

A first approximation to BW can be obtained by multiplying its inner surface area by its thickness T. We also know that BW is, necessarily, the same before and after voiding, so we have:


(2) $$4\pi r_{pre}^{2}\times T_{pre}=4\pi r_{post}^{2}\times T_{post}.$$

*V*_void_ is directly quantified in milliliters, and the pre and post-void thicknesses’ *T*_pre_ and *T*_post_ are ultrasonically measured (in centimeters). The solution set to equations 1 and 2 for *r*_pre_ and rpost has six roots, and only


(3) $$r_{pre}={\left(V_{void}\times \frac{3}{4\pi }\frac{T_{post}^{3/2}}{T_{post}^{3/2}-T_{pre}^{3/2}}\right)}^{1/3}{}_{,}$$

(4) $$r_{post}={\left(V_{void}\times \frac{3}{4\pi }\frac{T_{pre}^{3/2}}{T_{post}^{3/2}-T_{pre}^{3/2}}\right)}^{1/3}{}_{,}$$

is real and positive. Since


(5) $$T_{pre}\times \frac{T_{post}^{3/2}}{T_{post}^{3/2}-T_{pre}^{3/2}}=T_{post}\times {\frac{T_{pre}^{3/2}}{T_{post}^{3/2}-T_{pre}^{3/2}}}_{,}$$

**Figure 1:**
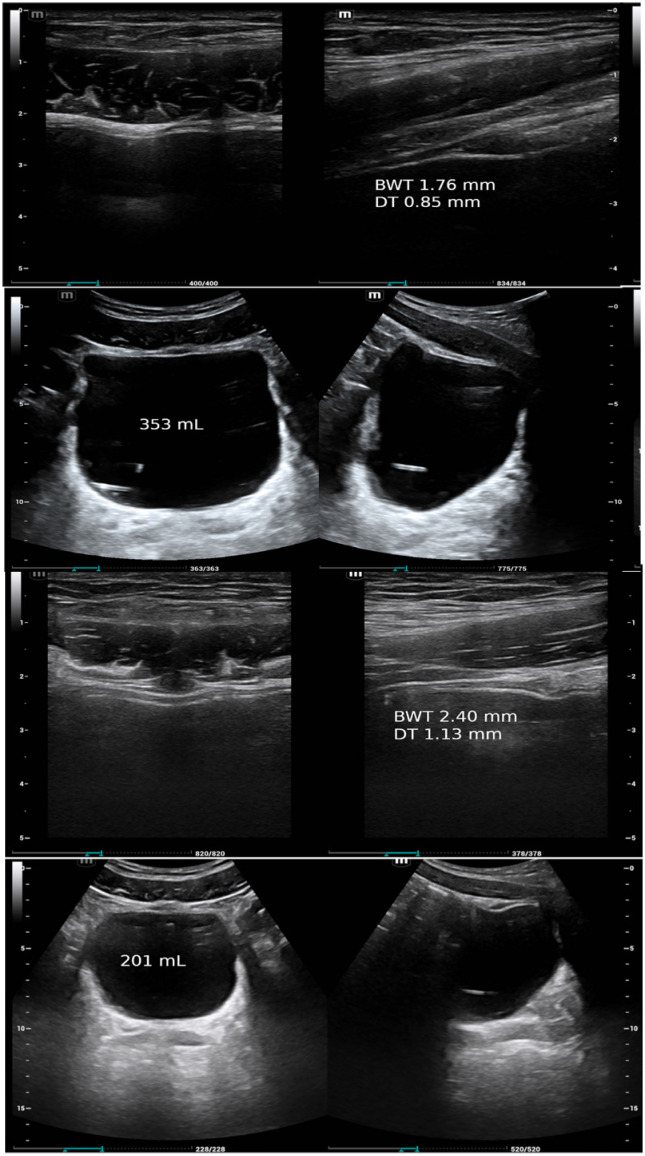
Ultrasonic images showing the layered aspect of the bladder wall in a patient with post-void residual urine (top images). Bladder and detrusor weight were calculated at 42.8 and 18.6 g by our method, compared to 42.3 and 19.3 g by Kojima's. Images obtained during cystometry with a 5 MHz curved array for whole bladder images and with a 10 MHz linear array for the bladder wall. From the first author's archives and used with permission. BWT, bladder wall thickness; DT, detrusor thickness.

One can use either solution, and either the left- or right-hand sides of equation 2 to write a formula for weight W, taking care to use the correct thickness in the calculation. As $3^{\frac{2}{3}}4^{\frac{1}{3}}\prod^{\frac{1}{3}}\approx 4.836$ we can use


(6) $$W_{bladder\text{,}detrusor}=4.836\times \frac{T_{post}T_{pre}V_{void}^{2/3}}{{\left(T_{post}^{3/2}-T_{pre}^{3/2}\right)}^{2/3}}$$

to calculate the weight *W* of the bladder or of the detrusor in grams. We have included an example, using archived images, comparing this method to Kojima's (Figure-1).

This method's main advantage is that it neither requires precise knowledge of intravesical volumes, nor, surprisingly, of post-void residual volumes – only the voided volume must be quantified – which enables its more widespread adoption. Still, as interesting and mathematically sound as this method is, we fully acknowledge its theoretical nature, and that it needs to undergo empirical scrutiny before integration into clinical practice.

Respectfully,

The Authors
